# Clinical and ultrasound features associated with congenital cytomegalovirus infection as potential predictors for targeted newborn screening in high-risk pregnancies

**DOI:** 10.1038/s41598-020-76772-1

**Published:** 2020-11-12

**Authors:** Hitomi Imafuku, Hideto Yamada, Akiko Uchida, Masashi Deguchi, Tokuro Shirakawa, Yuki Sasagawa, Yutoku Shi, Kazumichi Fujioka, Ichiro Morioka, Kenji Tanimura

**Affiliations:** 1 Department of Obstetrics and Gynecology, Kobe University Graduate School of Medicine, 7-5-1 Kusunoki-cho, Chuo-ku, Kobe, 650-0017 Japan; 2 Department of Pediatrics, Kobe University Graduate School of Medicine, Kobe, Japan; 3 Department of Pediatrics and Child Health, Nihon University School of Medicine, Tokyo, Japan

**Keywords:** Paediatric research, Microbiology, Diseases, Medical research, Risk factors

## Abstract

This prospective cohort study aimed to determine clinical factors associated with congenital cytomegalovirus (CMV) infection in pregnancy. Newborns born at a perinatal medical center received PCR analyses for CMV-DNA in their urine with informed consent. Clinical data, including age, maternal fever or flu-like symptoms, complications, ultrasound fetal abnormality, gestational weeks at delivery, and birth weight, were collected. Logistic regression analyses determined clinical findings associated with congenital CMV infection (cCMV). cCMV was diagnosed in 32 of 4380 pregnancies. Univariate and multivariable analyses revealed that age < 25 years old (OR 2.7, 95% CI 1.1–6.6; *p* < 0.05), the presence of maternal fever or flu-like symptoms (5.4, 2.6–11.2; *p* < 0.01), ultrasound fetal abnormalities (12.7, 5.8–27.7; *p* < 0.01), and preterm delivery at less than 34 gestational weeks (2.6, 1.1–6.0; *p* < 0.05) were independent clinical findings associated with cCMV. A combination of maternal fever/flu-like symptoms, ultrasound fetal abnormalities, or preterm delivery at less than 34 gestational weeks as optimal predictive factors showed 90.6% sensitivity, 66.4% specificity, and a maximum Youden index of 0.57. CMV-DNA tests in the urine of newborns born to mothers with these clinical manifestations may be an effective method in detecting cCMV as a targeted screening with a high sensitivity.

## Introduction

Cytomegalovirus (CMV) is the most common mother-to-child transmission in humans. Approximately 20% of neonates with cCMV are symptomatic. These clinical manifestations include fetal growth restriction (FGR), low birth weight (LBW), and central nervous system and multiple organ involvement with petechiae, hepatomegaly, splenomegaly, jaundice, pneumonia, and encephalitis. Infants with symptomatic cCMV develop neurological sequelae including hearing dysfunction, neuromuscular disorder, psychomotor retardation, ocular abnormality, delayed language development, and intellectual disability at incidences of 70–90%^1–3^. In addition, 10–15% of infants with asymptomatic cCMV develop long-term sequelae, such as progressive sensorineural hearing difficulty and mental retardation^2,4^.

Observational studies have shown higher incidences of cCMV in pregnancies with obstetric complications than in uncomplicated pregnancies. cCMV have been found in 1–4% of newborns with FGR, light-for-date, LBW, threatened premature labor, preterm delivery, and multiple pregnancy^5–8^. A prospective study involving 11,715 newborns screened by PCR tests for CMV-DNA in the saliva has found that socioeconomic factors such as younger age (< 25 years old), parous mothers born in high resources countries, and higher income are risk factors for cCMV due to maternal primary CMV infection^9^. On the other hand, younger and unemployed mothers are found to be risk factors for cCMV caused by non-primary CMV infection^9^. A recent study at a primary maternity hospital, where high-risk pregnancies are transferred to perinatal medical centers, has identified that maternal fever/flu-like symptoms or threatened miscarriage/premature labor in the second trimester are independent clinical findings predictive of the occurrence of cCMV^10^. However, pregnancies with severe hypertensive disorders of pregnancy (HDP), severe FGR, multiple pregnancy, fetal abnormality, threatened miscarriage/premature labor at hospitalization, or preterm delivery at less than 34 gestational weeks (GW) are excluded in the previous study^10^. These cases with severe complications have been transferred to a tertiary care perinatal medical center.

The aim of this prospective cohort study was to determine clinical findings associated with cCMV among women who gave birth at a tertiary care perinatal medical center, to which pregnant women with severe complications were referred or transferred.

## Methods

### Study design and participants

This prospective cohort study has been approved by the research ethics committee of Kobe University Graduate School of Medicine (reference number 923), and written informed consent was obtained from pregnant women. All research was performed in accordance with the relevant guidelines and regulations.

From February 2010 to August 2019, newborns born at the Kobe University Hospital, a perinatal medical center in Hyogo prefecture, Japan, underwent universal screening of polymerase chain reaction (PCR) tests for CMV-DNA in the urine. cCMV was diagnosed with the detection of CMV-DNA in their urine. All newborns who had positive results for CMV-DNA in the urine received a workup to identify symptoms of cCMV.

### Procedures

Pregnant women were inquired whether they had symptoms of fever, flu-like illness, genital bleeding, abdominal pain, uterine contraction and other abnormalities at the first visit and the regular prenatal checkup. Ultrasound examinations were performed by practiced obstetricians (H. I., K. T., M. D., or H. Y.) using the Voluson E8 Expert system (GE Healthcare, Milwaukee, Wisconsin) or the ProSound Alpha 7 system (Hitachi-Aloka Medical, Tokyo, Japan). They underwent measurements of estimated fetal weight by ultrasound at the regular prenatal checkup. The obstetricians collected the clinical data of pregnant women who visited to and gave birth at the university hospital, including age, gravidity and parity, fever or flu-like symptoms, obstetric and medical complications, delivery mode, gestational age at delivery, birth weight, and anomalies of newborns including cardiac malformation, renal dysplasia, intestinal atresia, and chromosomal abnormality. Women whose pregnancy ended in spontaneous miscarriages or stillbirth and women who gave birth at another hospital were excluded from study analyses.

The main reasons of referral to the university hospital were divided into four categories, including obstetric complications, medical complications, positive or borderline tests for CMV IgM, and none. The category of obstetrics complications included multiple pregnancies, threatened premature labor, FGR, ultrasound abnormality, low-lying placenta, placenta previa, hypertensive disorders of pregnancy, and gestational diabetes mellitus. The category of medical complications included diabetes mellitus, autoimmune disorders, antiphospholipid syndrome, thyroid diseases, heart disease, deep vein thrombosis, recurrent pregnancy loss, uterine myoma, epilepsy, and psychiatric disorders. In this study, fever or flu-like symptoms were defined as the condition where women complain of fever, nasal mucus, cough and/or sore throat. Threatened miscarriage and threatened premature labor were defined as the condition where women have subjective symptoms of uterine pain, contraction, bleeding, and/or shortening of uterine cervical length, and, therefore, require tocolytic agents, including oral administration of β-stimulant or calcium blocker, and intravenous administration of β-stimulant or magnesium sulfate for one or more weeks. FGR was defined as an estimated fetal body weight less than a mean − 1.5 standard deviation (SD) for gestational age. Microcephaly was defined as an occipitofrontal circumference less than a mean − 3.0 SD for gestational age. Light-for-date (LFD) was defined as a birth weight of less than the 10th percentile for gestational age. LBW was defined as a birth weight less than 2500 g.

Urine samples were collected from newborns on filter paper within one week after birth and the presence of CMV-DNA was assessed^11^; and cCMV was confirmed by PCR tests for the liquid urine samples^12^. Newborns with cCMV were diagnosed as symptomatic if they had at least one of the following symptoms: microcephaly, hepatosplenomegaly, hepatitis, thrombocytopenia, brain image abnormality, CMV associated retinopathy, or ABR abnormality^11,12^.

Pregnant women who visited the university hospital were asked to provide informed consent to undergo blood tests for CMV IgG and IgM, and these were measured among enrolled subjects until 22 GW. Pregnant women who were considered high risk for cCMV based on ultrasound abnormalities were recommended to have tests for CMV IgM after 22 GW. CMV IgG (negative, < 230; borderline, 230–240; and positive, > 240) was measured using the Enzygnost assay (Siemens Healthcare Diagnostics, Tokyo, Japan). CMV IgM (negative, < 0.8; borderline, 0.8–1.2; and positive, > 1.2 index) was measured using enzyme immunoassay kits produced by Denka Seiken (Tokyo, Japan).

From September 2013 to August 2019, newborns with informed consent from mothers received audiometry examinations of AABR screening tests for full-term newborns at 1–5 days in life or ABR tests for premature newborns before discharge. AABR abnormalities included a refer result whether unilateral or bilateral. ABR abnormalities whether unilateral or bilateral, included a non-response of V-wave to 40 dB for infants of postconceptional age 37 weeks and to 50 dB for infants of postconceptional age 34–36 weeks.

### Statistical analysis

Clinical characteristics were compared between pregnancies with and without cCMV. The differences between the two groups were analyzed using the Mann–Whitney U test, Fisher’s exact test, and the chi-squared test. Statistical significance was considered present at *p* values less than 0.05.

A stepwise approach was used to evaluate clinical findings associated with the occurrence of cCMV. Variables with *p* values less than 0.05 in univariate logistic regression analyses were subjected to multivariable logistic regression analyses, and variables with *p* values less than 0.05 in multivariable logistic regression analyses were determined as clinical findings significantly associated with the occurrence of cCMV. The optimal cutoff value was determined at the maximum Youden index. Sensitivity, specificity, positive predictive value (PPV), negative predictive value (NPV), and accuracy were calculated for the prediction of cCMV. All statistical analyses were performed using SPSS software, version 19 (SPSS Inc., Chicago, Illinois).

## Result

**Figure 1:**
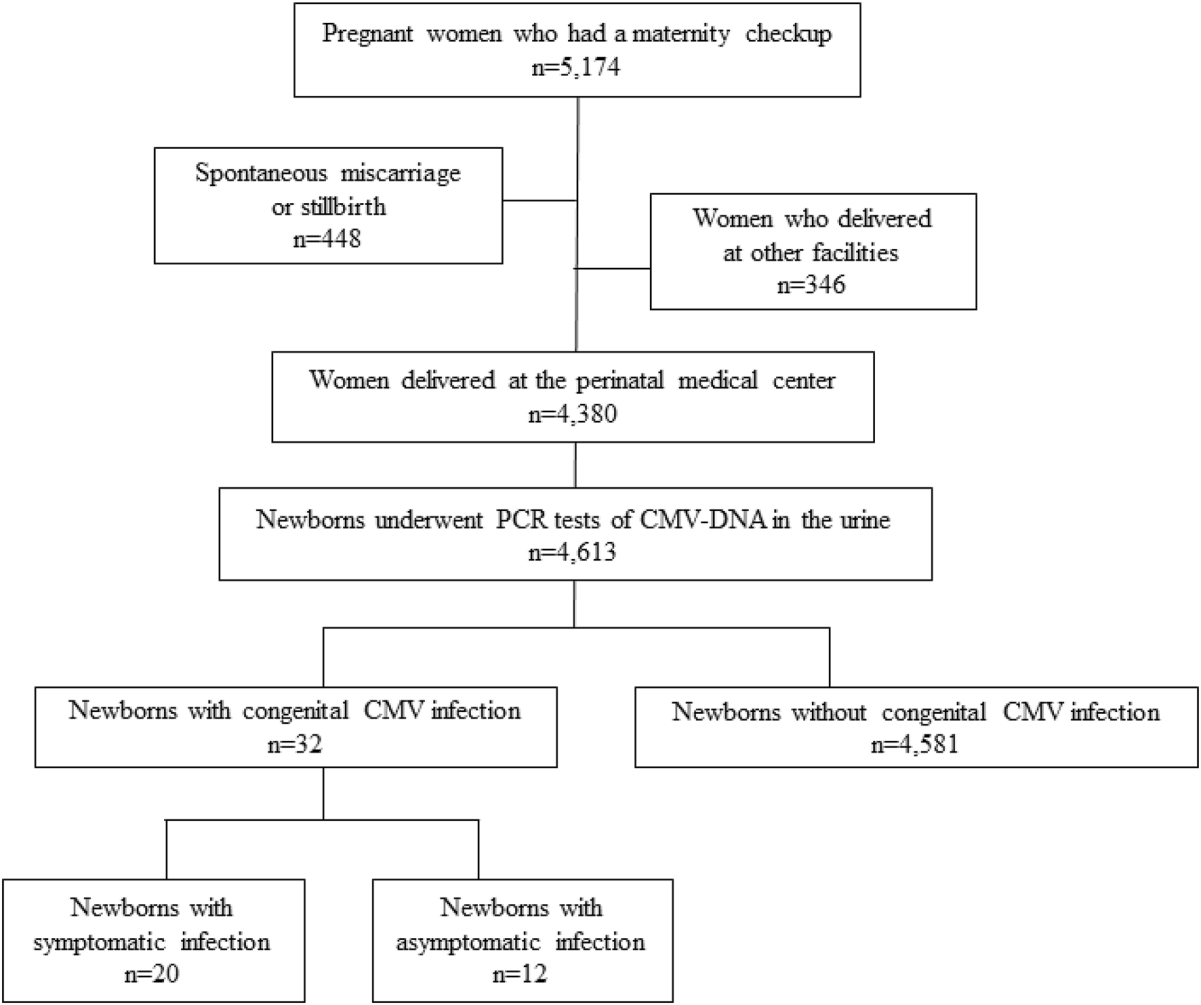
A flow diagram of study population and urine CMV screening of newborns. CMV, cytomegalovirus. During the study period, 5174 pregnant women visited to the university hospital. 448 pregnancies ended in spontaneous miscarriages or stillbirth, and 346 pregnant women delivered at other hospitals. A total of 4380 pregnant women who gave birth at the university hospital and their 4613 newborns who received PCR tests for CMV-DNA in the urine were enrolled. cCMV was diagnosed in 32 newborns (0.69%), including 20 newborns with symptomatic infection and 12 with asymptomatic infection.

A flow diagram of study population is shown in Fig. 1. During the study period, 5174 pregnant women visited to the Kobe University Hospital. Four hundred forty-eight pregnancies ended in spontaneous miscarriages or stillbirth, and 346 pregnant women delivered at other hospitals. A total of 4380 pregnant women and their 4613 newborns were enrolled. Of the 4380 pregnant women, 1392 (31.8%) had obstetrics complications, 1817 (41.5%) had medical complications, 1142 (26.1%) had advanced maternal age, 135 (3.1%) women had positive or borderline tests for CMV IgM, and 568 (13.0%) women without obstetric complications elected to deliver at the university hospital. cCMV was diagnosed in 32 newborns (0.69%), including 20 newborns with symptomatic infection and 12 with asymptomatic infection.

Table 1 shows clinical characteristics of the 4380 pregnant women. Women who had newborns with cCMV were significantly younger than those who had newborns without cCMV (*p* < 0.01). The frequencies of fever/flu-like symptoms (*p* < 0.01), FGR (*p* < 0.01), ultrasound fetal abnormalities including FGR (*p* < 0.01), preterm delivery at less than 37 weeks of gestation (GW) (*p* < 0.01), and preterm delivery at less than 34 GW (*p* < 0.01) were significantly higher than those in women who had newborns without cCMV. Gestational weeks at delivery in women who had newborns with cCMV were significantly earlier than those in women who had newborns without cCMV (*p* < 0.01). Table 2 shows clinical characteristics of the 4613 newborns. Birth weights of newborns with cCMV were significantly lower than those of newborns without cCMV (*p* < 0.01). The frequency of LBW in newborns with cCMV was significantly higher than that in newborns without cCMV (*p* < 0.01).

**Table 1:** Clinical characteristics of 4380 women.

Clinical findings of pregnant women	All women	Women who had newborns without congenital CMV infection	Women who had newborns with congenital CMV infection	*p* Value	Women who had newborns with symptomatic congenital CMV infection	Women who had newborns with asymptomatic congenital CMV infection	*p* Value
	n = 4380	n = 4348	n = 32		n = 20	n = 12	
Age, years old	33 (13–49)	33 (13–49)	29 (19–40)	< 0.01	29 (19–37)	30 (19–40)	N.S
Parity	0 (0–8)	0 (0–8)	0 (0–2)	N.S	0 (0–2)	0 (0–2)	N.S
Fever or flu-like symptoms	954 (21.8%)	935 (21.5%)	19 (59.4%)	< 0.01	13 (65.0%)	6 (50.0%)	N.S
Hypertensive disorders of pregnancy	359 (8.2%)	357	2	N.S	0	2	N.S
Gestational diabetes mellitus	347 (7.9%)	346	1	N.S	0	1	N.S
Diabetes mellitus	135 (3.1%)	134	1	N.S	0	1	N.S
Autoimmune disorders	166 (3.8%)	165	1	N.S	0	1	N.S
Thyroie diseases	252 (5.8%)	251	1	N.S	0	1	N.S
Multiple pregnancy	237 (5.4%)	234	3	N.S	1	2	N.S
Theretened miscarriafe or premature labor	1513 (34.5%)	1499	14	N.S	10	4	N.S
Fetal growth restriction	309 (7.1%)	301 (6.9%)	8 (25.0%)	< 0.01	7 (35.0%)	1 (8.3%)	N.S
Ultrasound fetal abnormalities	335 (7.6%)	316 (7.3%)	19 (59.4%)	< 0.01	17 (85.0%)	2 (16.7%)	< 0.01
Placenta previa	330 (7.5%)	330	0	N.S	0	0	N.S
Preterm delivery at less than 37 getational weeks	1138 (26.0%)	1118 (25.7%)	20 (62.5%)	< 0.01	17 (85.0%)	3 (25.0%)	< 0.01
Preterm delivery at less than 34 getational weeks	424 (9.7%)	413 (9.5%)	11 (34.4%)	< 0.01	10 (50.0%)	1 (8.3%)	< 0.05
Gestational weeks at delivery	38 (22–42)	38 (22–42)	36 (24–40)	< 0.01	34 (24–40)	39 (33–40)	< 0.01
Cesarean delivery	2168 (49.5%)	2147 (49.4%)	21 (65.6%)	N.S	17	4	< 0.01
CMV antibody tests until 22 weeks of gestation	n = 2295	n = 2287	n = 8		n = 2	n = 6	
Negative IgG	670 (29.2%)	669 (29.3%)	1 (12.5%)	N.S	0	1 (16.7%)	N.S
Positive IgG and negative IgM	1454 (63.4%)	1450 (63.4%)	4 (50%)	N.S	1 (50%)	3 (50%)	N.S
Positive IgG and positive IgM	171 (7.5%)	168 (7.3%)	3 (37.5%)	< 0.01	1 (50%)	2 (33.3%)	N.S
CMV antibody tests after 22 weeks of gestation	n = 222	n = 205	n = 17		n = 16	n = 1	
Negative IgG	49 (22.1%)	49 (23.9%)	0	< 0.01	0	0	N.S
Positive IgG and negative IgM	126 (56.8%)	126 (61.5%)	0	< 0.01	0	0	N.S
Positive IgG and positive IgM	47 (21.2%)	30 (14.6%)	17 (100%)	< 0.01	16 (100%)	1 (100%)	N.S

Data are expressed as median (range) or number (percentage).

CMV, cytomegalovirus; N.S., not significant.

**Table 2:** Clinical characteristics of 4613 newborns.

Clinical findings of newborns	All newborns	Newborns without congenital CMV infection	Newborns with congenital CMV infection	*p* Value	Newborns with symptomatic congenital CMV infection	Newborns with asymptomatic congenital CMV infection	*p* Value
	n = 4613	n = 4581	n = 32		n = 20	n = 12	
Male	2412 (52.3%)	2395	17	N.S	12	5	N.S
Birth weight, g	2824 (284–4748)	2830 (284–4748)	2252 (715–3320)	< 0.01	1904 (715–3216)	2819 (1744–3320)	< 0.01
Low birth weight	1394 (30.2%)	1375 (30.0%)	19 (59.4%)	< 0.01	16 (59.4%)	3 (59.4%)	< 0.01
Light-for-date	422 (10.9%)	416	6	N.S	5	1	N.S
Cardiac malformation	89 (1.9%)	88	1	N.S	0	1	N.S
Renal dysplasia	58 (1.3%)	58	0	N.S	0	0	N.S
Intestinal atresia	12 (0.3%)	12	0	N.S	0	0	N.S
Chromosomal abnormality	12 (0.3%)	12	0	N.S	0	0	N.S

Data are expressed as median (range) or number (percentage).

CMV, cytomegalovirus; N.S., not significant.

This cohort study revealed incidences of cCMV in pregnant women who had fever/flu-like symptoms (2.0%, 19/954), FGR (2.6%, 8/309), ultrasound fetal abnormalities including FGR (5.7%, 19/335), preterm delivery at less than 37 GW (1.8%, 20/1138), preterm delivery at less than 34 GW (2.6%, 11/424), and newborns with LBW (1.4%, 6/1394).

Table 3 shows ultrasound fetal abnormalities associated with cCMV in 4380 women. FGR (*p* < 0.01), ventriculomegaly (*p* < 0.01), microcephaly (*p* < 0.01), intracranial calcification (*p* < 0.01), pleural effusion (*p* < 0.05), ascites (*p* < 0.01), hepatosplenomegaly (*p* < 0.01), and hyperechoic bowel (*p* < 0.01) of the fetus in women who had newborns with cCMV were significantly higher than those in women who had newborns without cCMV.

**Table 3:** Ultrasound fetal abnormalities associated with congenital cytomegalovirus infection.

Ultrasound findings	All women	Women who had newborns without congenital CMV infection	Women who had newborns with congenital CMV infection	*p* Value	Women who had newborns with symptomatic congenital CMV infection	Women who had newborns with asymptomatic congenital CMV infection	*p* Value
	n = 4380	n = 4348	n = 32		n = 20	n = 12	
Fetal growth restriction	309 (7.1%)	301 (6.9%)	8 (25.0%)	< 0.01	7 (35.0%)	1 (8.3%)	N.S
Ventriculomegaly	14 (0.32%)	7 (0.02%)	7 (21.9%)	< 0.01	6 (30.0%)	1 (8.3%)	N.S
Microcephaly	33 (0.69%)	29 (0.7%)	4 (12.5%)	< 0.01	4 (20.0%)	0	< 0.01
Intracranial calcification	3 (0.07%)	0	3 (9.4%)	< 0.01	3 (15.0%)	0	< 0.01
Pleural effusion	4 (0.09%)	3 (0.07%)	1 (3.1%)	< 0.05	1 (5.0%)	0	< 0.05
Ascites	12 (0.27%)	4 (0.09%)	8 (25.0%)	< 0.01	8 (40.0%)	0	< 0.01
Hepatosplenomegaly	13 (0.30%)	2 (0.05%)	11 (34.4%)	< 0.01	11 (55.0%)	0	< 0.01
Hyperechoic bowel	8 (0.18%)	2 (0.05%)	6 (18.8%)	< 0.01	6 (30.0%)	0	< 0.01

Data are expressed as number (percentage).

Logistic regression analyses of clinical findings associated with cCMV during pregnancy were performed. Univariate logistic regression analyses for findings shown in Table 4 demonstrated that age < 25 years old (odds ratio [OR] 3.7, 95% confidence interval [CI] 1.6–8.5; *p* < 0.01), the presence of maternal fever or flu-like symptoms (OR 5.3, 95% CI 2.6–10.8; *p* < 0.01), FGR (OR 4.5, 95% CI 2.0–10.1; *p* < 0.01), ultrasound fetal abnormalities including FGR (OR 18.6, 95% CI 9.1–38.1; *p* < 0.01), preterm delivery at less than 34 GW (OR 5.0, 95% CI 2.4–10.4; *p* < 0.01), and LBW (OR 3.8, 95% CI 1.9–7.8; *p* < 0.01) were associated with the occurrence of cCMV. Preterm delivery at less than 34 GW but not at less than 37 GW was assessed because the former was clinically more important in perinatology. Eleven pregnancies with cCMV of fetuses ended in preterm delivery at less than 34 GW due to premature labor (n = 2), premature rupture of membranes (n = 2), severe FGR (n = 2), and severe fetal ascites (n = 2), polyhydramnios (n = 1), placental abruption (n = 1), and HELLP (hemolysis, elevated liver enzymes, low platelet count) syndrome (n = 1).

**Table 4:** Results of univariate and multivariable logistic regression analyses of clinical findings associated with congenital cytomegalovirus infection.

Clinical findings	Univariate analysis		Multivariable analysis	
	OR (95% CI)	*p* Value	OR (95% CI)	*p* Value
Age < 25 years old	3.7 (1.6–8.5)	< 0.01	2.7 (1.1–6.6)	< 0.05
Fever or flu-like symptoms	5.3 (2.6–10.8)	< 0.01	5.4 (2.6–11.2)	< 0.01
Fetal growth restriction	4.5 (2.0–10.1)	< 0.01		
Ultrasound fetal abnormalities	18.6 (9.1–38.1)	< 0.01	12.7 (5.8–27.7)	< 0.01
Preterm delivery at less than 34 getational weeks	5.0 (2.4–10.4)	< 0.01	2.6 (1.1–6.0)	< 0.05
Low birth weight	3.8 (1.9–7.8)	< 0.01		

OR, odds ratio.

Multivariable logistic regression analyses were performed for four clinical findings in pregnant women including age < 25 years old, the presence of maternal fever or flu-like symptoms, ultrasound fetal abnormalities including FGR, and preterm delivery at less than 34 GW (Table 4). It was revealed that age < 25 years old (OR 2.7, 95% CI 1.1–6.6; *p* < 0.05), the presence of maternal fever/flu-like symptoms (OR 5.4, 95% CI 2.6–11.2; *p* < 0.01), ultrasound fetal abnormalities (OR 12.7, 95% CI 5.8–27.7; *p* < 0.01), and preterm delivery at less than 34 GW (OR 2.6, 95% CI 1.1–6.0; *p* < 0.05) were independent clinical findings associated with the occurrence of cCMV in pregnant women who delivered at a perinatal medical center.

The optimal clinical findings associated with cCMV were assessed using the maximum value of the Youden index which is defined as “sensitivity + specificity − 1.” Combination of the presence of maternal fever/flu-like symptoms, ultrasound fetal abnormalities, or preterm delivery at less than 34 GW were determined as optimal predictive factors, showing sensitivity of 90.6%, specificity of 66.4%, positive predictive value of 1.9%, negative predictive value of 99.9%, accuracy of 66.6%, and a maximum Youden index of 0.57 (Table 5).

**Table 5:** Optimal clinical findings associated with congenital cytomegalovirus infection.

Factors	Sensitivity, %	Specificity, %	Positive Predictive	Negative Predictive	Accuracy, %	Yoden
			Value, %	Value, %		Index
Age < 25	21.9	92.9	2.2	99.4	92.4	0.15
Fever/flu-like symptoms	59.4	78.5	2.0	99.6	78.4	0.38
Ultrasound fetal abnormalities	59.4	92.7	5.7	99.7	92.5	0.52
Preterm delivery at less than 34 gestational weeks	34.4	90.5	2.6	99.5	90.1	0.25
Fever/flu-like symptoms or ultrasound fetal abnormalities	84.4	72.9	2.3	99.8	73.0	0.57
Fever/flu-like symptoms or preterm delivery at less than 34 gestational weeks	75.0	70.1	1.8	99.7	70.2	0.45
Ultrasound fetal abnormalities or preterm delivery at less than 34 gestational weeks	68.8	85.5	3.4	99.7	85.4	0.54
Fever/flu-like symptoms or ultrasound fetal abnormalities or preterm delivery at less than 34 gestational weeks	90.6	66.4	1.9	99.9	66.6	0.57
Age < 25 or fever/flu-like symptoms or ultrasound fetal abnormalities or preterm delivery at less than 34 gestational weeks	90.6	62.1	1.7	99.9	62.3	0.53

In 32 newborns with cCMV, 6 newborns (18.8%) were likely born to mothers with non-primary CMV infection, since all the 6 mothers had positive CMV IgG and negative CMV IgM. Two of the six newborns had symptomatic cCMV. All six pregnant women were referred to the university hospital due to obstetrics complications (n = 2), medical complications (n = 3) and none of referral reason (n = 1). The other 26 newborns (81.2%) with cCMV were likely born to mothers with primary CMV infection during pregnancy, since all the 26 mothers had positive CMV IgM. Eighteen of the 26 newborns had symptomatic cCMV. Sixteen of the 26 pregnant women were referred to the university hospital due to positive or borderline tests for CMV IgM. The remaining 10 pregnant women were referred due to ultrasound fetal abnormalities (n = 6), obstetric complications (n = 3), and medical complications (n = 2).

From September 2013 to August 2019, 2814 newborns received automated auditory brainstem response (AABR)/auditory brainstem response (ABR) screening tests. Eight of 18 (44.4%) newborns with cCMV and 71 of 2796 (2.5%) newborns without cCMV had abnormality of AABR/ABR tests. Abnormality of AABR/ABR tests for newborns in women who had newborns with cCMV were significantly higher than those in women who had newborns without cCMV (*p* < 0. 01).

## Discussion

This cohort study at a perinatal medical center demonstrated that clinical findings including age younger than 25 years, the presence of fever/flu-like symptoms, FGR, ultrasound fetal abnormalities, premature delivery, earlier gestational weeks at delivery in pregnant women; lighter birth weight and LBW in newborns were associated with cCMV. Abnormality of AABR/ABR tests in newborns was also associated with cCMV. Univariate and multivariable logistic regression analyses for the first time determined that maternal age less than 25 years old, the presence of fever/flu-like symptoms, ultrasound fetal abnormalities, and premature delivery at less than 34 GW in pregnant women were independent clinical findings predictive of the occurrence of cCMV in this cohort study. In addition, a combination of the presence of maternal fever/flu-like symptoms, ultrasound fetal abnormalities, or preterm delivery at less than 34 GW were determined as optimal predictive clinical factors, showing the highest sensitivity of 90.6% and a maximum Youden index of 0.57. If women have these clinical findings during pregnancy, they should receive serological tests of CMV IgG/IgM together with CMV-DNA test in the urine of their newborns due to a higher risk for cCMV. Newborns with LBW or abnormality of AABR/ABR tests should also receive CMV DNA test in the urine. An early diagnosis of cCMV can lead to an early commencement of antiviral therapies for infants with symptomatic cCMV to reduce neurological impairments and sequelae^13^.

This cohort study also revealed high incidences of cCMV in pregnant women who had fever/flu-like symptoms (2.0%), FGR (2.6%), ultrasound fetal abnormalities including FGR (5.7%), preterm delivery at less than 37 GW (1.8%), preterm delivery at less than 34 GW (2.6%), and newborns with LBW (1.4%). This information will be useful for obstetricians and neonatologists in considering risks of cCMV at perinatal medical centers. The incidence of cCMV (0.69%) in the present study was relatively higher than that of previous reports in Japan (0.31%–0.46%)^5,14^, because the subjects included high-risk pregnancies.

Maternal age at less than 25 years old was identified as an independent risk factor for cCMV in the present study, and this was coincident with the results of a European study^9^. The ultrasound fetal abnormalities including FGR during pregnancy was also identified as a risk factor for cCMV. Similarly, several studies have reported that ultrasound fetal abnormalities and/or FGR during pregnancy are associated with newborns with cCMV^5,15–17^.

An observational study has shown higher frequencies of maternal fever/flu-like symptoms in pregnant women with CMV infection compared with controls^18^. The presence of maternal fever/flu-like symptoms had been revealed as a risk factor for cCMV in a cohort study for low-risk pregnancies at a primary maternity hospital^10^. The present cohort study for high-risk pregnancies at a perinatal medical center also identified maternal fever/flu-like symptoms as an independent risk factor for cCMV. Maternal fever/flu-like symptoms may be associated with primary or reinfection of CMV during pregnancy, causing CMV transmission to their fetuses. Alternatively, maternal infection of other microorganisms causing fever/flu-like symptoms may reactivate latent CMV infection in the uterus and blood circulation, causing CMV transmission to their fetuses. CMV may reach the placenta by viremia, or by ascending route from the uterine cervix^19^.

Preterm delivery at less than 34 GW was identified as an independent risk factor for cCMV. An observational study has shown that 3.0% of women with preterm delivery at less than 37 GW bore newborns with cCMV^8^; and a cohort study for low-risk pregnancies at a primary maternity hospital has revealed that threatened premature labor in the second trimester is a risk factor for cCMV^10^. Severe cases with threatened premature labor may be transferred from primary maternity hospitals to perinatal medical centers as high-risk pregnancies, and these pregnancies may result in preterm delivery at less than 34 GW. Preterm delivery may be caused by intrauterine CMV infection as an effect of cCMV. Alternatively, preterm delivery may be associated with inflammation causing reactivation of latent CMV in the uterus and blood circulation.

CMV-DNA tests in the urine for newborns born to mothers who have fever/flu-like symptoms, ultrasound fetal abnormalities, or preterm delivery may be an effective method in detecting cCMV as a targeted screening with a high sensitivity at perinatal medical centers. Newborns with LBW or abnormality of AABR/ABR tests should receive CMV-DNA test in the urine. These results will provide useful information for clinical practitioners to consider risks of cCMV at perinatal medical centers. The findings in the present study should be followed by further studies in other population.

## Data Availability

The datasets analyzed during the current study are available from the corresponding authors on reasonable request.

## References

[1] Revello MG, Gerna G (2002). Diagnosis and management of human cytomegalovirus infection in the mother, fetus, and newborn infant. Clin. Microbiol. Rev..

[2] Stagno S, Whitley RJ (1985). Herpesvirus infections of pregnancy. Part I: Cytomegalovirus and Epstein-Barr virus infections. New Engl. J. Med..

[3] Pass RF, Stagno S, Myers GJ, Alford CA (1980). Outcome of symptomatic congenital cytomegalovirus infection: results of long-term longitudinal follow-up. Pediatrics.

[4] Stagno S (1986). Primary cytomegalovirus infection in pregnancy: incidence, transmission to fetus, and clinical outcome. JAMA.

[5] Tanimura K (2017). Universal screening with use of immunoglobulin G avidity for congenital cytomegalovirus infection. Clin. Infect. Dis..

[6] Vaudry W (2010). Congenital cytomegalovirus infection in high-risk Canadian infants: report of a pilot screening study. Can. J. Infect. Dis. Med. Microbiol..

[7] van der Weiden S (2011). Is routine TORCH screening and urine CMV culture warranted in small for gestational age neonates?. Early Hum. Dev..

[8] Lorenzoni F (2014). Neonatal screening for congenital cytomegalovirus infection in preterm and small for gestational age infants. J. Maternal-fetal Neonatal Med. Off. J. Eur. Assoc. Perinatal Med. Fed. Asia Ocean. Perinatal Soc. Int. Soc. Perinatal Obstet..

[9] Leruez-Ville M (2017). Risk factors for congenital cytomegalovirus infection following primary and nonprimary maternal infection: a prospective neonatal screening study using polymerase chain reaction in saliva. Clin. Infect. Dis..

[10] Uchida A (2019). Clinical factors associated with congenital cytomegalovirus infection: a cohort study of pregnant women and newborns. Clin. Infect. Dis..

[11] Kobayashi Y (2015). Low total IgM values and high cytomegalovirus loads in the blood of newborns with symptomatic congenital cytomegalovirus infection. J. Perinatal Med..

[12] Matsuo K (2014). Quantitative evaluation of ventricular dilatation using computed tomography in infants with congenital cytomegalovirus infection. Brain Dev..

[13] Kimberlin DW (2015). Valganciclovir for symptomatic congenital cytomegalovirus disease. New Engl. J. Med..

[14] Koyano S (2011). Screening for congenital cytomegalovirus infection using newborn urine samples collected on filter paper: feasibility and outcomes from a multicentre study. BMJ Open.

[15] Guerra B (2008). Ultrasound prediction of symptomatic congenital cytomegalovirus infection. Am. J. Obstet. Gynecol..

[16] Enders G, Bader U, Lindemann L, Schalasta G, Daiminger A (2001). Prenatal diagnosis of congenital cytomegalovirus infection in 189 pregnancies with known outcome. Prenat. Diagn..

[17] Sonoyama A (2012). Low IgG avidity and ultrasound fetal abnormality predict congenital cytomegalovirus infection. J. Med. Virol..

[18] Nigro G, Anceschi MM, Cosmi EV (2003). Clinical manifestations and abnormal laboratory findings in pregnant women with primary cytomegalovirus infection. BJOG.

[19] Nigro G (2011). Role of the infections in recurrent spontaneous abortion. J. Maternal-fetal Neonatal Med. Off. J. Eur. Assoc. Perinat. Med. Fed. Asia Ocean. Perinat. Soc. Int. Soc. Perinat. Obstet..

